# Association of endothelial nitric oxide synthase gene variants with preeclampsia

**DOI:** 10.1186/s12978-021-01213-9

**Published:** 2021-07-28

**Authors:** Ghazala Shaheen, Sarwat Jahan, Nousheen Bibi, Asmat Ullah, Rani Faryal, Ali Almajwal, Tayyaba Afsar, Dara Al-disi, Mahmoud Abulmeaty, Abdulaziz Abdullah Al Khuraif, Mohammed Arshad, Suhail Razak

**Affiliations:** 1 Department of Animal Sciences, Faculty of Biological Sciences, Quaid-I-Azam University, Islamabad, 45320 Pakistan; 2 Department of Bioinformatics, Shaheed Benazir Bhutto Women University, Peshawar, Pakistan; 3 College of Applied Medical Sciences, Community Health Sciences, King Saud University, Riyadh, Kingdom of Saudi Arabia; 4 Department of Biochemistry, Faculty of Biological Sciences, Quaid-I-Azam University, Islamabad, 45320 Pakistan; 5 Department of Microbiology, Faculty of Biological Sciences, Quaid-I-Azam University, Islamabad, 45320 Pakistan; 6 Dental Biomaterials Research Chair, Dental Health Department, College of Applied Medical Sciences, King Saud University, Riyadh, Kingdom of Saudi Arabia

**Keywords:** Preeclampsia, Endothelial nitric oxide synthase gene, Nitric oxide, Variants

## Abstract

**Background:**

Preeclampsia (PE) is a complex pregnancy hypertensive disorder with multifaceted etiology. The endothelial nitric oxide synthase (*eNOS*) gene and nitric oxide (NO) levels has been reported to be associated with PE predisposition in various populations. Therefore, present study was designed to investigate the role of NO levels and *eNOS* gene variants in preeclamptic women in Pakistan.

**Methods:**

A total of 600 women were evaluated, 188 of PE with mild features, 112 of PE with severe features and 300 normotensive pregnant women. NO levels were detected by Greiss reaction method and genotyping following sequencing was conducted for *eNOS* gene variants. Further *insilico* studies were performed to get insights into the structural and functional impact of identifies mutation on eNOS protein as well as on protein regulation.

**Results:**

Reduced concentrations of NO were reported in all PE groups (p < 0.05) as compared to controls. The frequency of c.894 T (p.298Asp) and g.-786C alleles were significantly associated with PE. In addition, novel homozygous variant g.2051G > A was also significantly associated with PE when compared to normotensive women. Dynamic simulation studies revealed that Glu298Asp mutation destabilize the protein molecule and decrease the overall stability of eNOS protein. Molecular docking analysis of mutant promoter with transcription factors STAT3 and STAT6 proposed changes in protein regulation upon these reported mutations in upstream region of the gene.

**Conclusion:**

Considering the results of current study, the functional alterations induced by these variants may influence the bioavailability of NO and represents a genetic risk factor for increased susceptibility to PE. However, large studies or meta-analysis are necessary to validate these findings.

**Supplementary Information:**

The online version contains supplementary material available at 10.1186/s12978-021-01213-9.

## Plain Language Summary

Preeclampsia (PE) is a complex pregnancy hypertensive disorder with multifaceted etiology characterized by increased hypertension and proteinuria after 20 weeks of gestation. The present study was directed to determine the role of *eNOS* in susceptibility to PE and the association of c.894G > T (p.(Glu298Asp), intron 4b/4a, g.-786 T > C and other possible variants of *eNOS* gene with preeclampsia in Pakistani population. Computational analysis of identified variants in the coding and non-coding region of the *eNOS* gene was also conducted to determine the change in gene regulation and further protein stability. A total of 600 women were evaluated, 188 with mild and 112 with PE with severe features PE with 300 normotensive pregnant women. NO levels and genotyping following sequencing was conducted for *eNOS* gene variants. Further *insilico* studies were performed to get insights into the structural and functional impact of identifies mutation on eNOS protein as well as on protein regulation. Data from the current study suggest that there might be other risk variants of the *eNOS* gene (g.2051G > A and g.1861G > A) and lower levels of serum NO that confers in an increased risk of PE. The detailed computational investigation further confirmed the deformities and changes in protein flexibility upon Glu298Asp. These structural alterations might be associated with preeclampsia. Variants in the promoter region of the *eNOS* gene further validate the change in gene regulation for the onset of disease. Identification of key structural and functional features in eNOS protein and gene regulatory region might be used for designing specific drugs for therapeutic purpose.

**Supplementary Information:**

The online version contains supplementary material available at 10.1186/s12978-021-01213-9.

## Background

Preeclampsia (PE) is a complex pregnancy hypertensive disorder with multifaceted etiology characterized by de novo hypertension, with or without proteinuria, accompanied with the signs of maternal acute kidney injury (AKI), neurological features, liver dysfunction, hemolysis or thrombocytopenia, or fetal growth restriction after 20 weeks of gestation [1–3]. It is the leading cause of maternal and prenatal morbidity and mortality in developing countries [4]. Annually 63,000 maternal and 50,000 infant’s deaths are predicted to be associated with PE [5–7]. Prevalence of PE is higher in developing countries like Pakistan where PE and eclampsia was around 19% and 1 in 89 women dies of maternal causes [8, 9]. Studies have shown various factors involved in the susceptibility of PE, but etiological details are still being debated [10]. Pathogenesis of PE is multifactorial involving environmental and genetic factors [11]. It was determined that defective placentation is associated with the progression of PE [12]. Disturbed trophoblastic invasion and spiral arteries remodeling results in endothelial dysfunction which leads to vasoconstriction [11].

Various mediators are involved in controlling endothelial dysfunction in PE but the role of endothelial nitric oxide synthase (*eNOS*) gene located at the 7q35-q36 region appears most significant in the development of PE [13]. *eNOS* is an important regulator of vascular tone and contributes to the reduction of the uteroplacental resistance seen in normal pregnancy through the production of Nitric Oxide (NO) by reduction of L-arginine to L-citruline [14]. Polymorphisms of *eNOS* impair NO availability which is crucial for maternal vascular vasodilation during pregnancy, leading to susceptibility of PE [15, 16].

Several variants in *eNOS* gene were identified effecting its functions or production levels (Rahimi et al., 2013). These variants comprise of functional c.894G > T (p.(Glu298Asp)) variant in exon 7 of *eNOS* gene.[16–19], an insertion-deletion variant in intron 4 (4b/4a) consisting of 27 bp tendem repeats, 4b allele comprises of 5 repeats and 4a allele with 4 repeats [4, 10, 20] and g.-786 T > C variant present in promotor region of *eNOS* gene[1, 15, 21].

Considering the potent role of polymorphism in the *eNOS* gene in causing clinical symptoms of PE different mega projects and meta-analysis were conducted to better understand the underlying mechanisms but there are many gaps to be filled [22]. Under a detailed study projected towards this important issue related to maternal health, for the first time the role of *eNOS* gene in PE was highlighted in Pakistan. The present study was directed to determine the role of *eNOS* in susceptibility to PE and the association of c.894G > T (p.(Glu298Asp), intron 4b/4a, g.-786 T > C and other possible variants of *eNOS* gene with PE in Pakistani population. Computational analysis of identified variants in the coding and non-coding region of the *eNOS* gene was also conducted to determine the change in gene regulation and further protein stability (Additional file 1).

## Methods

### Compliance

The present study was conducted with prior approval from ethical committees of Quaid-i-Azam University, Islamabad (BEC-FBS-QAU2015-98) and collaborating hospitals including Pakistan Institute of Medical Sciences (PIMS) (F.1-1/2015/ERB/SSZABMU), Islamabad and Quaid-e-Azam International Hospital, Islamabad (QAIH-2015/11). All participants were informed about the study objectives and signed an informed consent. The study protocol was done in accordance with the principles of the Declaration of Helsinki.

### Participants

The studied individuals comprised of 300 pregnant women with preeclampsia including 188 with mild features and 112 with severe features and 300 normotensive pregnant women as controls which were recruited at Pakistan Institute of Medical Sciences (PIMS) and Quaid-e-Azam International Hospital, Islamabad during the period of September 2015 to July 2017. These are the important hospitals in the capital territory of Pakistan where patients visit from different rural and urban areas and they provide the quality health services with personalized and specialized care for patients. According to the hospital staff 1 in 10 pregnant women is diagnosed with hypertension which also include PE. That’s why prominent number of deliveries occur in the respective hospitals. All women included were < 35 years of age. Informed consent and a detailed Performa (including history and clinical examination) were filled before sample collection. Blood pressure of all study subjects was measured by sphygmomanometer in millimeters of mercury (mmHg).

### Inclusion and exclusion criteria

The control group have women with uncomplicated gestation and blood pressure < 125/85 mmHg and no proteinuria. Diagnostic criteria for PE with mild features was blood pressure ≥ 140/90 mmHg, and proteinuria ≥  + 1 on Dipstick test. PE with severe features was defined as blood pressure ≥ 160/110 mmHg, and proteinuria (proteinuria ≥  + 3 on Dipstick test [23]. Exclusion criteria involved diabetes, asthma, kidney disease, hematological disorder, autoimmune disease, current or history of smoking and eclampsia.

### Sampling and storage

Blood was collected during the antepartum period (third semester, 36.32 ± 0.36 weeks for controls 33.14 ± 0.48 for PE with mild features, 33.08 ± 0.56 for PE with severe features and 33.12 ± 0.15 for total PE group) before the onset of delivery in labelled tubes for clinical investigations and genetic analysis. Blood samples were divided into two parts, serum was separated immediately by centrifugation at 3000 rpm for 10 min from half of the blood in non-EDTA tubes and stored at − 80 °C for estimation of NO, while second part present in EDTA tubes was stored at − 4 °C for DNA extraction, genotyping and sequencing.

### Genetic analysis

DNA was extracted from blood using GF-1 Blood DNA Extraction Kit (Vivantis Technology, USA) according to manufacturer’s instruction. Primers (humanizing genomics macrogen, Korea) were selected from the previous study [24]. c.894G > T (p.(Glu298Asp) (rs1799983) variant in exon 7 of the *eNOS* gene was detected by Polymerase chain reaction (PCR) (Maxygene Thermal Cycler 230v, TWN) using the forward primer of 5ʹ-CATGAGGCTCAGCCCCAGAAC-3ʹ and reverse primer of 5ʹ-AGTCAATCCCTTTGGTGCTCAC-3ʹ. After amplification the PCR product was digested using 5U MboI restriction endonuclease enzyme (Cat#ER0811 Thermo Scientific, USA) by overnight incubation at 37 °C. Genotypes were determined by separation of fragments on 2% agarose gel.

Intron 4b/4a (rs1722009) allele was detected by PCR method using a forward primer of 5ʹ-AGGCCCTATGGTAGTGCCTTG-3ʹ and reverse primer of 5ʹ-TCTCTTAGTGCTGTGGTCAC-3ʹ. The PCR product was electrophoresed on 2.5% agarose gel.

g.-786 T > C (rs2070744) variant in promoter region of *eNOS* gene was detected by Tetra ARMS-PCR method using an outer forward primer of 5ʹ-TTTCTCCAGCCCCTCAGATG-3ʹ and outer reverse primer of 5ʹ-AGGCCCAGCAAGGATGTAGT-3ʹ, while inner primer for C allele was 5ʹ-CATCAAGCTCTTCCCTGGCC-3ʹ and inner primer for T allele was 5ʹ-CATCAAGCTCTTCCCTGGCT-3ʹ respectively. The target gene was amplified, the double reaction was conducted for each sample with respective inner allele primer and the PCR product was subjected to 2% agarose gel electrophoresis. For the confirmation of these variants and other new variants in the respective regions Sanger sequencing was performed (DTCS Quick Start Kit; Beckman Coulter, Fullerton, CA, USA).

### Estimation of NO

Nitric oxide (NO) decomposes from nitrite (NO_2_^−^) and nitrate (NO_3_^−^). Nitrite is the stable end product and estimated as an index of NO by using Greiss reaction. For the quantitative determination of NO, total NO kit (Cat# EMSNO, Thermo Scientific, USA) was used according to the manufacturer’s instruction in which enzyme nitrate reductase converts nitrate to nitrite. Nitrite is then detected as a colored azo dye product of the Griess reaction that absorbs visible light at 540 nm. The interaction of NO in a system was measured by the determination of total nitrate and nitrite concentrations in the sample.

### Computational analysis

#### In silico site directed mutagenesis of *eNOS* protein

The 3D structure of mutant (*eNOS*^Glu298Asp^) protein was built in Chimera using normal eNOS protein 3D structure (PDBID: 1m9q) as template. The stereochemical properties and environmental profile of the mutant model was evaluated using PROCHECK [25] and ERRAT (Structure analysis and verification servers) respectively. Mutant model was minimized using Chimera 1.5.6 [26] and NOMAD-Ref (http://lorentz.immstr.pasteur.fr/nomad-ref.php) further the root-mean-square deviation (RMSD) of native and mutant structure was also calculated.

#### Molecular dynamic simulations

Molecular dynamic (MD) simulations studies were of *eNOS*^WT^ and mutant *eNOS*^Glu298Asp^ were made to assess the folding, stability,, conformational changes and dynamic behaviors of eNOS3 protein. Amber03 force field embedded in GROMACS 4.5 package [27] running on high performance OpenSuse linux system was used to perform simulations. Throughout simulations experiments, both eNOS3^WT^ and mutant *eNOS*^Glu298Asp^ systems were solvated by TIP4P [28] water model in a periodic box. The system was neutralized by addition of Na^+^ and Cl^ˉ^ counter ions. Energy minimization (steepest descent algorithm for 500 steps) was executed by the tolerance of 1000 kJ/mol Å^2^ to eliminate initial steric clashes. After completing the minimization steps systems were subjected to simulations for 20 ns time scale under constant temperature (300 K) and pressure (1 atm). To this end electrostatic interactions were calculated using Particle Mesh Ewald (PME) algorithm. To investigate the stability behavior of *eNOS*^WT^ and mutant *eNOS*^Glu298Asp^ systems, VMD [29], PyMol (http://www.pymol.org) and GROMACS tools were used.

#### Transcription factor binding site prediction in the promoter region

Transcription factors are key to the regulation of genes and form the basis of gene regulation studies and understanding of gene on and off mechanism. ConTraV3 (http://bioit2.irc.ugent.be/contra/v3) and JASPER database were used for the prediction of transcription factor binding site in the promoter region of *eNOS* gene.

#### DNA model building

DNA model was generated for promoter region with three sequences (Seq1: CCCTCAGATGGCACAGAACTAC, Seq2: CTTCCCTGGCTGGCTGACCCTG, Seq3: CCCGGGAAGCGTGCGTCACTG) of *eNOS* Nucleic Acid Builder (NAB) program [30, 31] and 3D-Dart server [32]. All the generated models were minimized for phosphate backbone geometry optimization.

#### Protein DNA molecular docking

Protein-DNA interactions are key to in several biological processes, DNA repair, gene regulation and chromatin structural organization. DOT 2.0 program suite [33] (http://www.sdsc.edu/CCMS/DOT) was used to calculate the potential binding site and binding energies of the promoter region of *eNOS* and its putative transcription factor protein. Grid was set large enough for smooth movement of molecule around the stationary molecule. Additionally, at the grid boundaries stationary potential was near zero to reduce the artifacts from fast Fourier calculations. In present docking experiments rotational sets of 28,800 (7.5°) and 54,000 (6°) were used.

### Statistical analysis

All data were expressed as mean ± S.E.M (standard error of the mean). Statistical analysis for NO levels was carried out by using lme4 [34] and easyanova [35] package of R 3.5.1 (R Development Core Team, 2018) and NO concentrations were compared with age, gestational age and BMI by analysis of variance. The difference between groups was analyzed by ANOVA ea1 command and graph was obtained by ggboxplot command in ggpubr package of R. Statistical analysis of clinical data and the frequency of alleles and genotypes were compared between study and control group by Independent sample t test, Chi-squared test (χ^2^) and Odds Ratio (OR) with values predicted by Hardy–Weinberg equilibrium model and linear regression by using IBM SPSS Statistics 21 software. Values of p < 0.05 were considered statistically significant.

## Results

### Characteristics of study individuals

The characteristics of cases and controls at diagnosis are shown in Table 1. The significant difference was noted between control and preeclamptic groups with regard to age (p ≤ 0.001 and p = 0.002), body mass index (BMI) (p ≤ 0.001 and p = 0.047), systolic and diastolic blood pressure (p ≤ 0.001), Gestational age (p ≤ 0.001), proteins in urine (p ≤ 0.001) and child weight at birth (p ≤ 0.001). Furthermore, the family history of preeclampsia and preeclampsia in previous pregnancy and in primigravida was more prominent in the preeclamptic groups as compared to normotensive females (Table 2).

**Table. 1:** Clinical characteristics of controls and Preeclamptic groups

**Parameters**	**Controls (n = 300)**	**PE with mild features (n = 188)**	**PE with severe features (n = 112)**	**Total preeclamptic women (n = 300)**
Age (Years)	27.017 ± 0.32	28.14 ± 0.42 p ≤ 0.001	29.17 ± 0.59 p ≤ 0.001	28.52 ± 0.34 0.002
BMI (kg/m^2^)	27.67 ± 0.28	28.72 ± 0.47 p ≤ 0.001	28.39 ± 0.54 p ≤ 0.001	28.59 ± 0.360 0.047
Gestational age (Weeks)	36.32 ± 0.36	33.14 ± 0.48 p ≤ 0.001	33.08 ± 0.56 p ≤ 0.001	33.12 ± 0.15 p ≤ 0.001
Systolic Blood Pleasure (mmHg)	114.71 ± 0.51	40.39 ± 0.93 p ≤ 0.001	174.94 ± 2.02 p ≤ 0.001	154.36 ± 1.54 p ≤ 0.001
Diastolic Blood Pleasure (mmHg)	73.88 ± 0.45	93.10 ± 0.86 p ≤ 0.001	112.51 ± 1.86 p ≤ 0.001	100.95 ± 1.12 p ≤ 0.001
Proteinuria	0.08 ± 0.03	2.1 ± 0.04 p ≤ 0.001	2.49 ± 0.06 p ≤ 0.001	2.29 ± 0.05 p ≤ 0.001
Infant’s weight (g)at birth	3029.45 ± 31.31	2600 ± 130 p ≤ 0.001	22,100 ± 190 p ≤ 0.001	2412.5 ± 21.06 p ≤ 0.001

All data are reported as mean ± standard deviation with p = p value, BMI, Body mass index

**Table. 2:** Pregnancy history of control group and preeclamptic groups

Parameters	Controls (n = 300)	PE with mild features (n = 188)	PE with severe features (n = 112)	Total preeclamptic women (n = 300)	Total (n = 600)
Gravidity Primary Gravida Multiple Gravida	177 (59) 123 (41)	145 (77.13) 43 (22.87) p ≤ 0.001	80 (71.43) 32 (28.57) p ≤ 0.001	225 (75) 75 (25) p ≤ 0.001	402 (67) 198 (33)
Preeclampsia in previous pregnancy Yes No	15 (5) 285 (95)	56 (29.79) 132 (70.21) p ≤ 0.001	28 (25) 84 (75) p ≤ 0.001	84 (28) 216 (72) p ≤ 0.001	99 (16.5) 501 (83.5)
Preeclampsia in Family Yes No	12 (4) 288 (96)	43 (22.87) 145 (77.13) p ≤ 0.001	35 (31.25) 77 (68.75) p ≤ 0.001	78 (26) 222 (76) p ≤ 0.001	90 (15) 510 (85)

### Estimation of NO

**Fig. 1:**
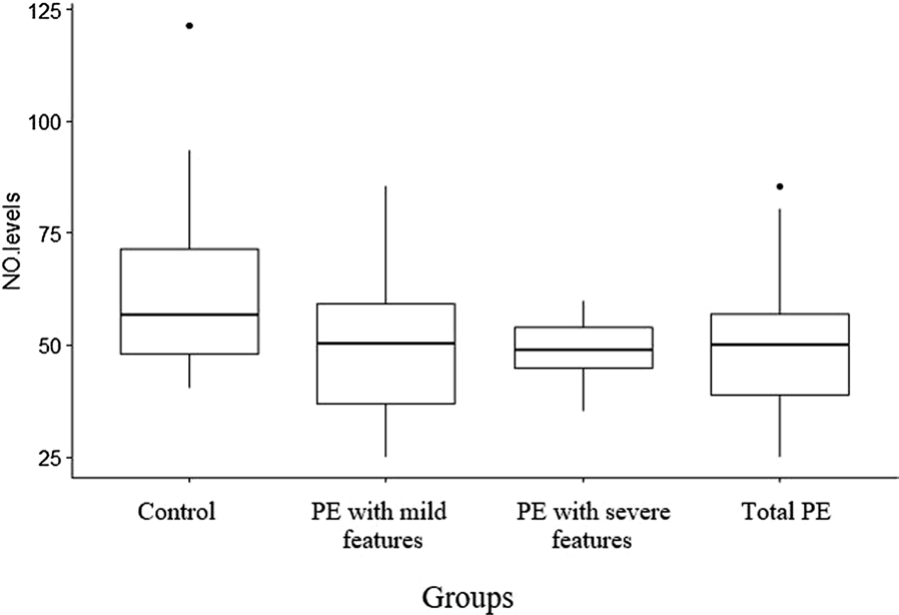
Concentration of NO in controls and preeclamptic group

Serum NO levels were estimated in all study groups comparable with normotensive healthy controls and difference between them was significant (F = 4.87, P = 0.003). Significantly low levels of NO were observed in PE with mild features (50.18 ± 2.78, P = 0.003), PE with severe features (48.59 ± 3.68, P = 0.02) and total preeclamptic group (49.6 ± 2.22, p = 0.002) as compared to control group (62.12 ± 2.88) (Fig. 1). When NO levels were compared to age, gestational age and BMI the results were non-significant with p value of 0.14, 0.52 and 0.40.

### eNOS variants distribution

**Fig. 2:**
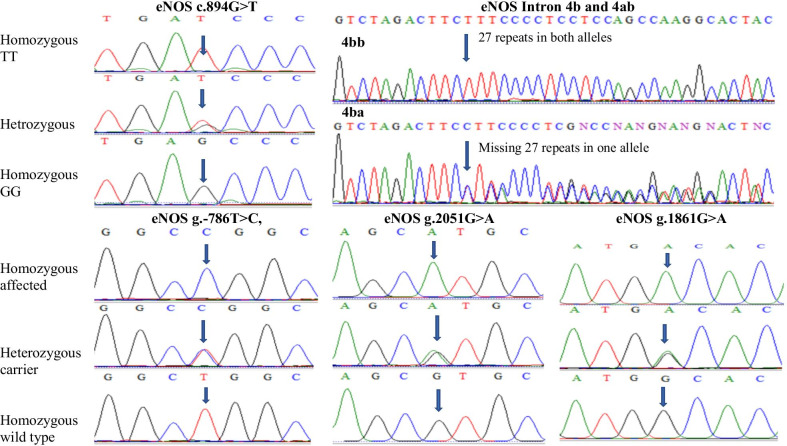
Sequence analysis of eNOS gene variants

The distribution of *eNOS* c.894G > T (p.(Glu298Asp)), intron 4b/4a and g.-786 T > C variants were studied in all groups as presented in Table 3, Fig. 2. The genotypic distribution was in Hardy–Weinberg equilibrium. For c.894G > T (p.(Glu298Asp)) variant electrophoresis of digested product yield band sizes of 206 bp for GG, 206 bp/119 bp/87 bp for GT and 119 bp/87 bp for TT genotype respectively. Frequency of GG, GT and TT genotypes was 66.4%, 12.3% and 21.3% in PE with mild features, 65.2%, 12.5% and 22.3% in PE with severe features and 66%, 12% and 22% in total preeclamptic women as compared to controls (75%, 14.5% and 10.5%) respectively. While frequency G and T alleles was 72.3% and 27.7% in PE with mild features, 71.4% and 28.6% in PE with severe features while 72% and 28% in total preeclamptic individuals as compared to control group (82.25% and 17.75%) respectively. Significant difference in frequency of c.894 T allele in PE with mild features (p ≤ 0.001), severe (p = 0.004) and total preeclamptic women (p ≤ 0.001) was observed likewise c.894TT (p.298AspAsp) genotype showed higher frequency in PE with mild features preeclamptic individuals (p = 0.02), preeclamptic group with severe features (p = 0.03) and total preeclamptic women (p = 0.008) as compared to control group.

**Table. 3:** Genotype distribution of *eNOS* 894 G/T, Intron 4b/4a, -786 T/C, -2051 G/A and -1861G/A haplotypes in controls and preeclamptic groups

*eNOS* Variants	Genotype and Alleles	Controls (total n = 300) n (%)	PE with mild features (total n = 188) n (%)	PE with severe features (total n = 112) n (%)	Total preeclamptic women (total n = 300) n (%)
c.894G > T or p.(Glu298Asp)	GG	225 (75)	125 (66.4)	73 (65.2)	198 (66)
rs1799983	TC	43 (14.5)	23 (12.3)	14 (12.5)	36 (12)
	TT	32 (10.5)	40 (21.3)	25 (22.3)	66 (22)*
			(χ^2^ = 7.56, p = 0.023)	(χ^2^ = 6.79, P = 0.033)	(χ^2^ = 9.75, P = 0.008)
	Allele Frequency				
	G (Glu)	493 (82.25)	272 (72.3)	160 (71.4)	432 (72)
	T (Asp)	107 (17.75)	104 (27.7)	64 (28.6)	168 (28)
			(χ^2^ = 22.78 p ≤ 0.001)	(χ^2^ = 7.91, p = 0.004)	(χ^2^ = 11.91, p ≤ 0.001)
			OR = 2.27 (1.61–3.2)	OR = 1.862 (1.20–2.88)	OR = 1.80 (1.28–2.52)
Intron 4a/4b	Bb	272 (90.5)	159 (84.6)	99 (88)	258 (86)
rs1722009	Ba	28 (9.5)	29 (15.4)	13 (12)	42 (14)
	Aa	0	0	0	0
			(χ^2^ = 2.42, p = 0.08)	(χ^2^ = 0.37, p = 0.34)	(χ^2^ = 1.95, p = 0.1)
	Allele Frequency				
	B	572 (95.25)	347 (92.3)	210 (94)	558 (93)
	A	28 (4.75)	29 (7.7)	14 (6)	42 (7)
			(χ^2^ = 2.51, p = 0.08)	(χ^2^ = 0.43, p = 0.32)	(χ^2^ = 2.07, p = 0.09)
			OR = 1.69 (0.87–3.26)	OR = 1.31 (0.58–2.97)	OR = 1.54 (0.85–2.82)
g.-786 T > C	TT	204 (68)	107 (56.9)	63 (56)	169 (56.5)
rs2070744	TC	79 (26.5)	61 (32.4)	45 (40)**	104 (34.5)**
	CC	17 (5.5)	20 (10.7)*	4 (4)	27 (9)*
			(χ^2^ = 7.19, P = 0.02)	(χ^2^ = 7.24, P = 0.02)	(χ^2^ = 7.84, P = 0.02)
	Allele Frequency				
	T	487 (81.25)	272 (72.3)	170 (76)	442 (73.75)
	C	113 (18.75)	104 (27.7)	54 (24)	158 (26.25)
			(χ^2^ = 6.98, p = 0.006)	(χ^2^ = 1.86, p = 0.1)	(χ^2^ = 6.45, p = 0.007)
			OR = 1.65 (1.13–2.40)	OR = 1.36 (0.87–2.14)	OR = 1.54 (1.10–2.15)
g.-2051G > A	GG	230 (76.5)	117 (62.3)	79 (70.5)	196 (65.5)
rs553827594	GA	51 (17)	36 (19.1)	10 (9)	47 (15.5)
	AA	19 (6.5)	35 (18.6)**	23 (20.5)**	57 (19)***
			(χ^2^ = 12.19, P = 0.002)	(χ^2^ = 12.16, P = 0.002)	(χ^2^ = 7.45, P = 0.02)
	Allele Frequency				
	G	511 (85)	270 (72)	168 (75)	439 (73.25)
	A	89 (15)	106 (28)	56 (25)	161 (26.75)
			(χ^2^ = 16.25, p < 0.001)	(χ^2^ = 7.01, p = 0.008)	(χ^2^ = 6.45, p = 0.007)
			OR = 2.10 (1.49–3.25)	OR = 1.85 (1.16–2.44)	OR = 1.85 (1.17–2.94)
g.-1861G > A	GG	212 (71)	123 (65.4)	78 (69.7)	201 (67)
rs1800779	GA	83 (27.5)	59 (31.2)	26 (23.2)	85 (28.5)
	AA	5 (1.5)	6 (3.2)	8 (7.1)*	14 (4.5)
			(χ^2^ = 1.72, P = 0.42)	(χ^2^ = 5.27, P = 0.07)	(χ^2^ = 3.26, P = 0.19)
	Allele Frequency				
	G	507 (84.75)	305 (81.2)	182 (81.25)	487 (81.25)
	A	93 (15.25)	71 (18.8)	42 (18.75)	113 (18.75)
			(χ^2^ = 1.39, p = 0.23)	(χ^2^ = 0.93, p = 0.33)	(χ^2^ = 1.73, p = 0.18)
			OR = 1.28 (0.84–1.95)	OR = 1.27 (0.77–2.08)	OR = 1.28 (0.88–1.85)

*p ≤ 0.05 and **p ≤ 0.01 is significant for Pearson chi-square test to identify which genotype presents significant different frequencies

As a result of electrophoresis *eNOS* 4b allele produced band of 420 bp and 4ba allele produced bands of 4420 bp and 393 bp. Intron 4bb and 4ba genotypes were found in 84.6% and 15.4% individuals of PE with mild features, 88% and 12% subjects of severe features however 86% and 14% women of total preeclamptic group as compared to control subjects (90.5% and 9.55). aa genotype was found in none of the patients or control subjects. Frequency of b and a allele was 92.3% and 7.7% in PE with mild features, 94% and 6% in PE with severe features group while 93% and 7% in total preeclamptic individuals as compared to control group (95.25% and 4.75%). Frequency of intron 4b/4b and intron 4b/4a showed no significant difference in all three groups (p = 0.08, p = 0.32 and p = 0.09) as compared to normotensive pregnant females.

Electrophoresis for g.-786 T > C variant showed first band of 387 bp which was control band in both PCR reaction (T and C) and second band of 250 bp, corresponds to T and C allele indicating homozygous genotype (TT and CC), and heterozygous genotype (TC) in case of both bands in both reactions. Genotypic distribution of g.-786TT, TC and CC was 56.9%, 32.4% and 10.7% in PE with mild features, 56%, 40% and 4% in PE with severe features however 56.5%, 34.5% and 9% in total preeclamptic women when compared to control group (68%, 26.5% and 5.5%). The frequency of g.-786C allele was significantly different in PE with mild features (p = 0.006) and total preeclamptic group (p = 0.007) as compared to healthy controls. Likewise, g.-786TC genotype determined significant difference in PE with severe features (p < 0.01) and total preeclamptic group (p < 0.01) when compared to normotensive control group. G.-786CC genotype showed significant difference in PE with mild features (p < 0.05) and total preeclamptic group (p < 0.05) as compared to control group.

Other variants g.-2051G > A (rs553827594, in novel homozygous AA genotype) and g.-1861G > A (rs1800779) were also found in preeclamptic women in homozygous GG, heterozygous GA and homozygous AA genotypes. g.-2051G > A showed significant association with all PE (p = 0.02) groups as compared to control group, while the non-significant association of g.-1861G > A was found between groups as shown in Table 2 and Fig. 2. Which may have a possible association with preeclampsia.

### Structural analysis

**Fig. 3:**
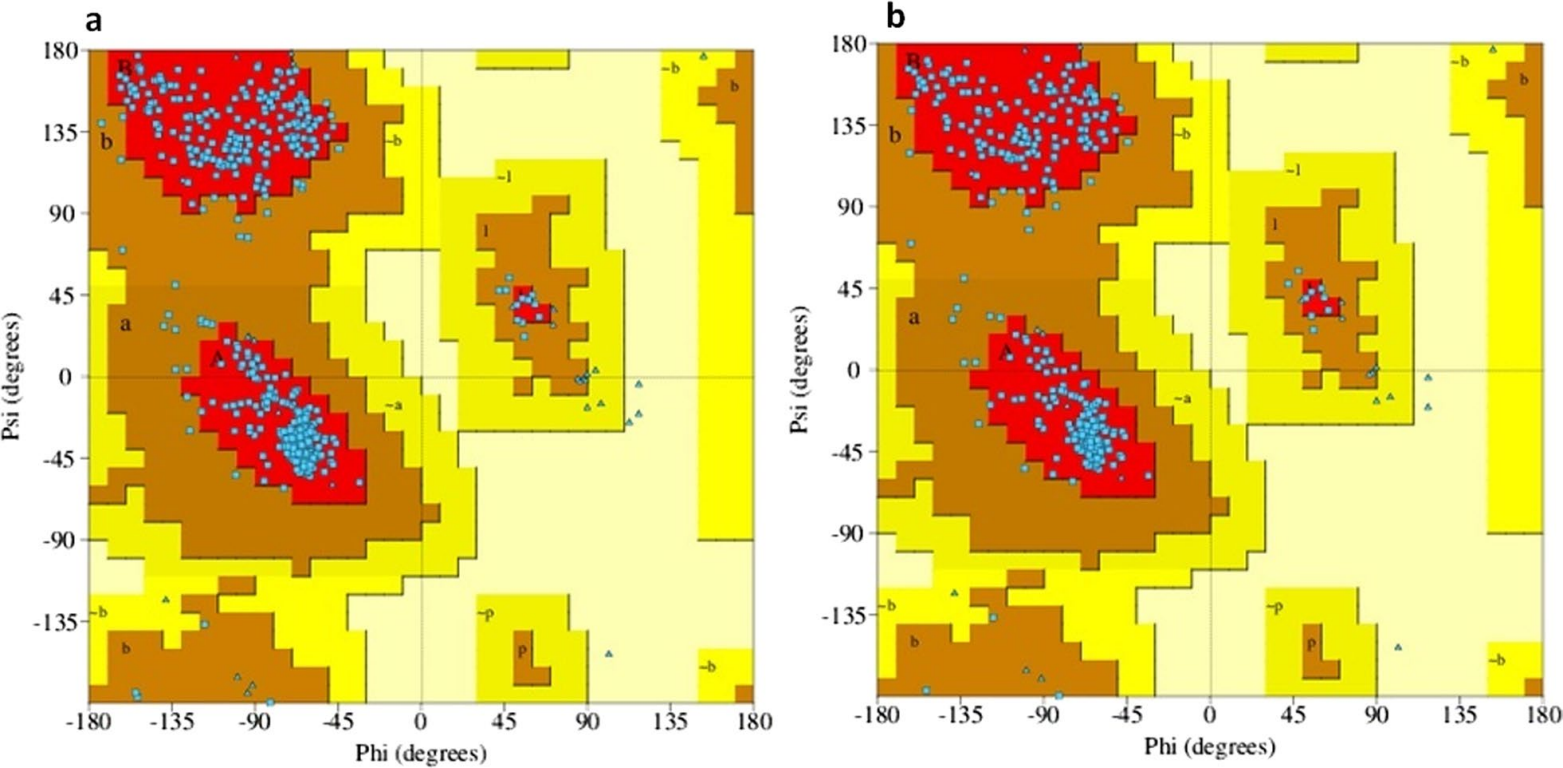
Ramachandran plot. **a** eNOS^WT^, **b** eNOS^Glu298Asp^

**Fig. 4:**
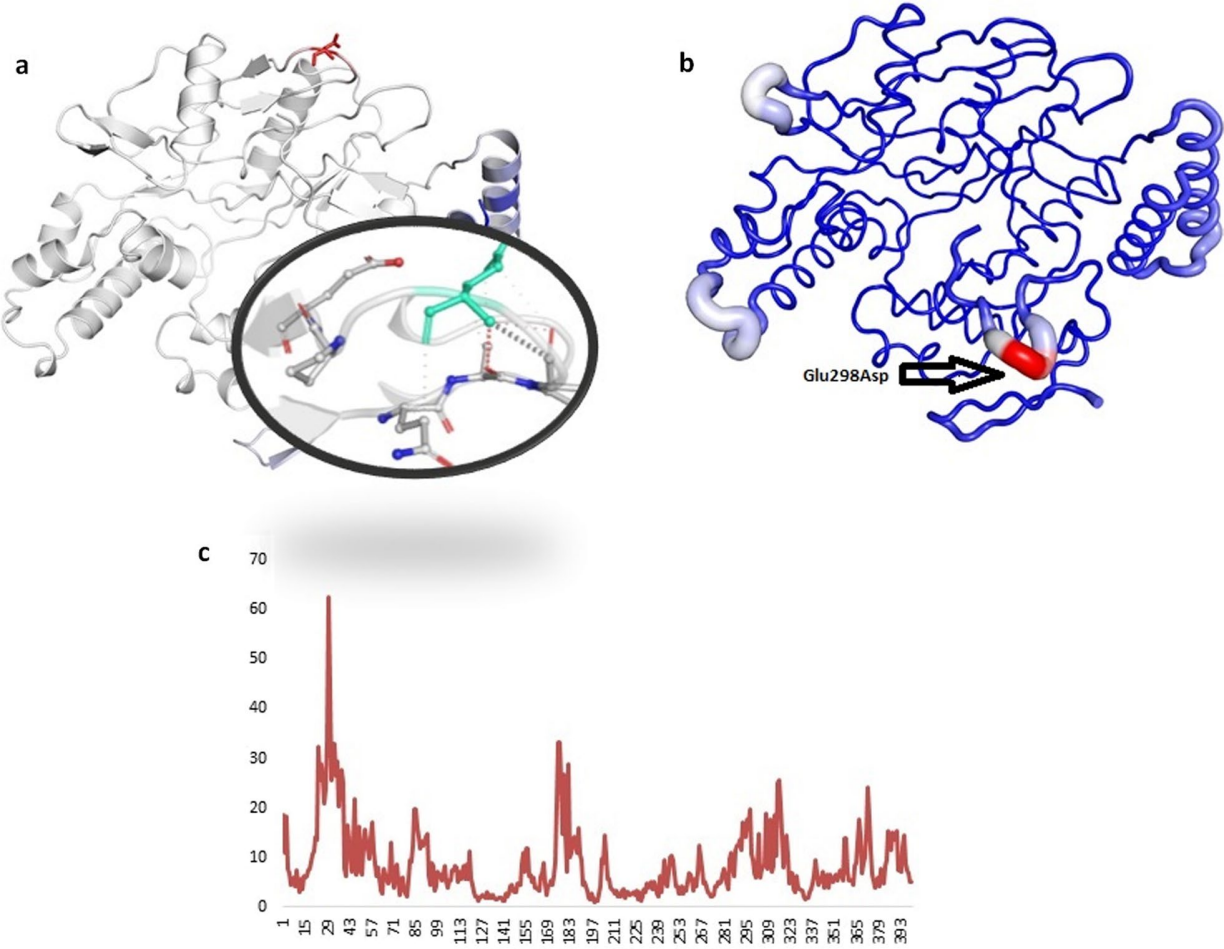
Structural changes upon Glu298Asp substitution in *eNOS* protein. **a** eNOS^WT^ protein, **b **eNOS^MT^ with red colored tube represent the magnitude of fluctuation and deformation upon mutation. Amino acids colored according to the vibrational entropy change upon mutation. blue represents a rigidification of the structure and red a gain in flexibility (**c**) shows the atomic representation of substitution of Glu to Asp amino acid substituent indicated in cyan color. **c** Plot showing deformities in protein upon mutation

Ramachandran plot for *eNOS*^Glu298Asp^ structure indicated overall > 93% of residues in the allowed region (Fig. 3). This shows that structure is of good quality and can be used for the structure–activity study. Structural analysis revealed the fact that this substitution changed the overall topology of the eNOS protein. Upon mutation stability of the protein changes with destabilizing energy of ΔΔG: − 0.071 kcal/mol. The flexibility of the protein was also changed with vibrational entropy change of ΔΔS_Vib_ ENCoM: − 0.396 kcal.mol^−1^.K^−1^. Several atomic fluctuations were observed throughout the eNOS protein (Fig. 4).

### Molecular dynamics simulation analysis

**Fig. 5:**
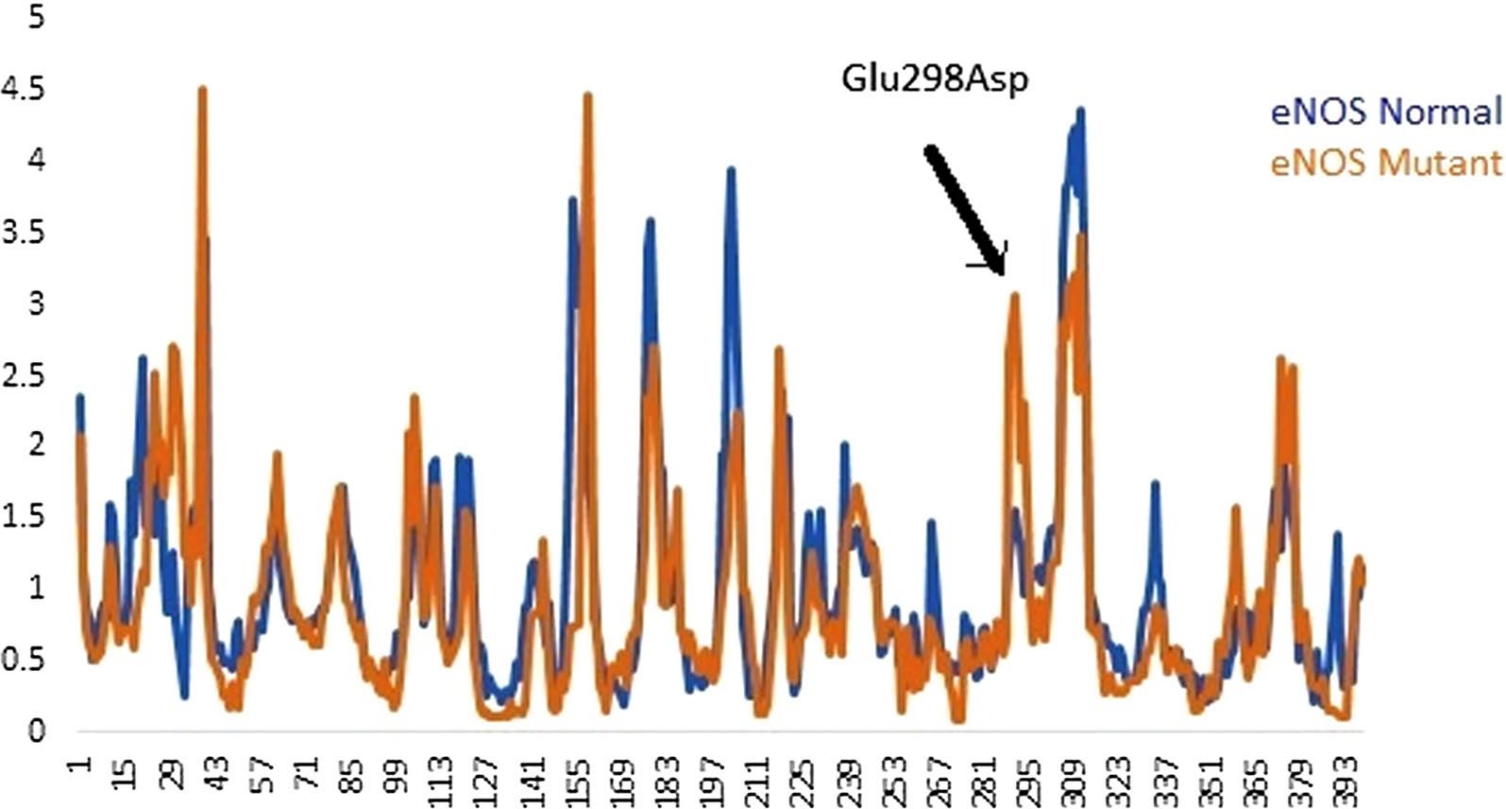
Root Mean Square Fluctuation plot of *eNOS* normal and mutant protein

The *eNOS*^WT^ and *eNOS*^Glu298Asp^ structures were further analyzed by molecular dynamics (MD) simulation assay to study the time-dependent behavior and overall stability of the system. secondary structural elements stability and conformational deviations of all atom trajectories were used to plot the root mean square fluctuation (RMSF) and root mean square deviation (RMSD). In *eNOS*^WT^ the high fluctuation was observed between 150–300 residues and the systems remain stable throughout the protein length. The RMSF analysis of eNOS^Glu298Asp^ indicated fluctuation in the loops near mutant residue at 298 position. The high RMSF fluctuation rate of the *eNOS*^Glu298Asp^ per residue indicated high fluctuation rate in the C-terminus regions of the protein. To our surprise region encompassing the substitution Glue298Asp indicated more fluctuation upto 5A^º^. This high fluctuation indicated that this substitution has negative impact on protein stability (Fig. 5).

### Transcription factor binding Site prediction and DNA modeling

**Fig. 6:**
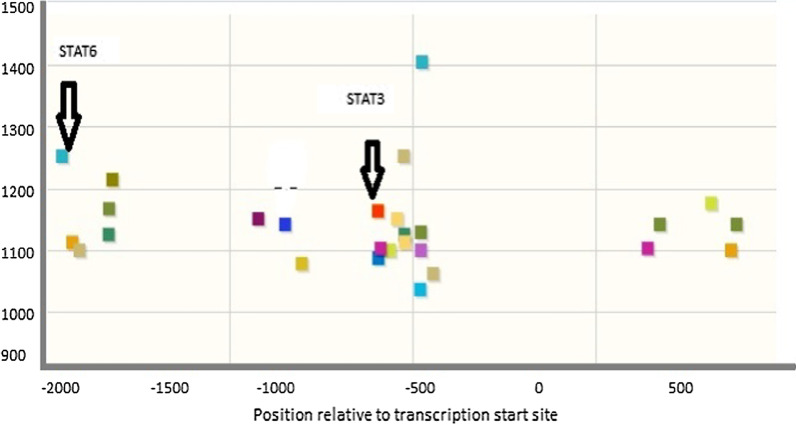
Scatter plot of TF binding sites on eNOS promoter

**Fig. 7:**
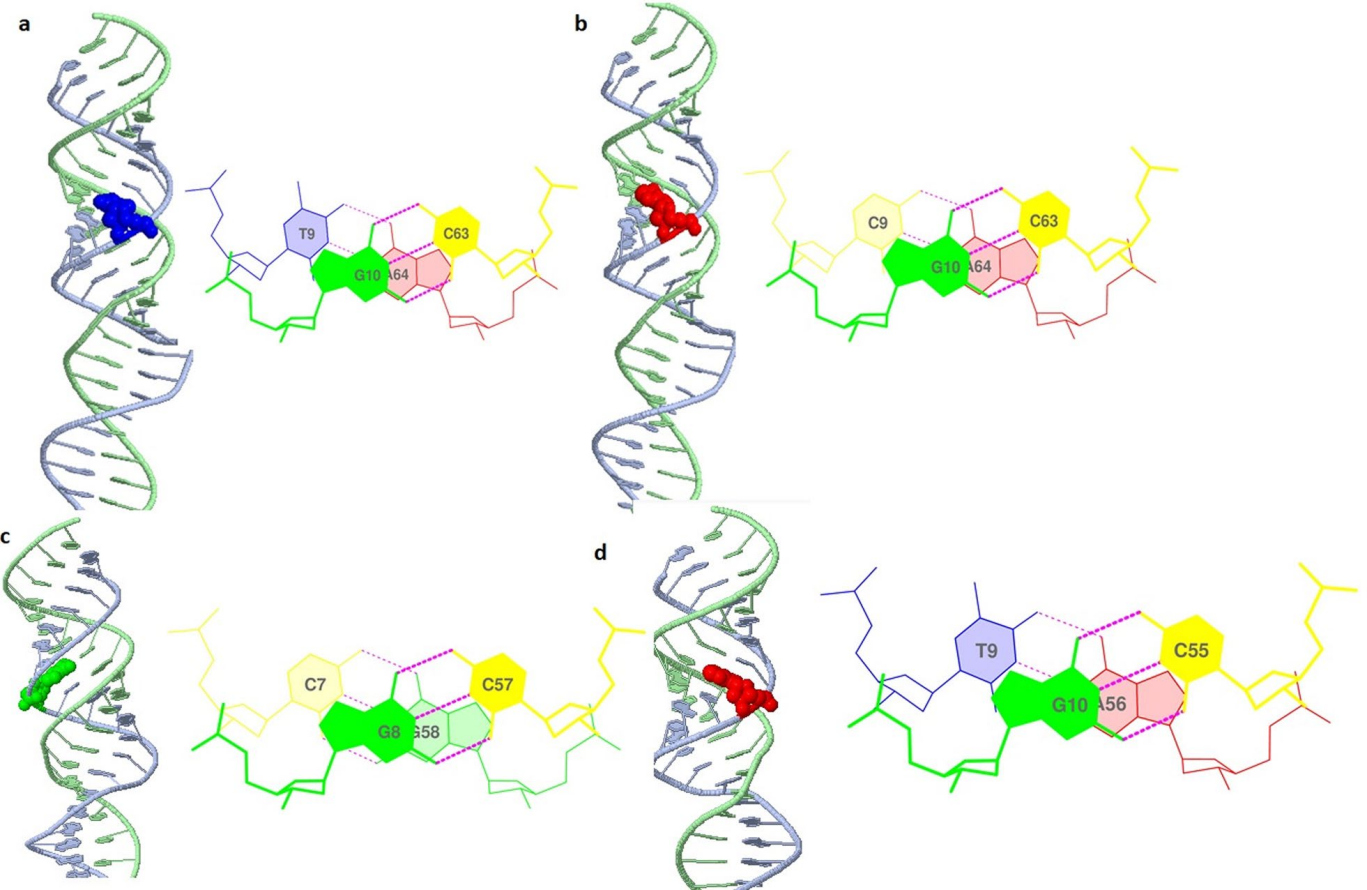
DNA modeling and stacking interaction of normal and mutant DNA molecules

ConTraV3 (http://bioit2.irc.ugent.be/contra/v3) and JASPER database revealed Signal transducer and activator of transcription 3 (STAT 3) and Signal transducer and activator of transcription 6 (STAT6) transcription binding site in the promoter region of *eNOS* gene (Fig. 6). STAT3 is a transcription factor involved in cell proliferation, inflammation, differentiation, and survival (Qi QR and Yang ZM., 2014). STAT6/IL-4 pathway is important for normal pregnancy (KR bound., 2017). Variants in the promoter region (− 786 T > C and − 2015G > A) abolished the binding site for Stat3 and Stat 6 respectively. Normal and mutant DNA molecules were modeled for the promoter region of *eNOS* gene (Fig. 7).

**Fig. 8:**
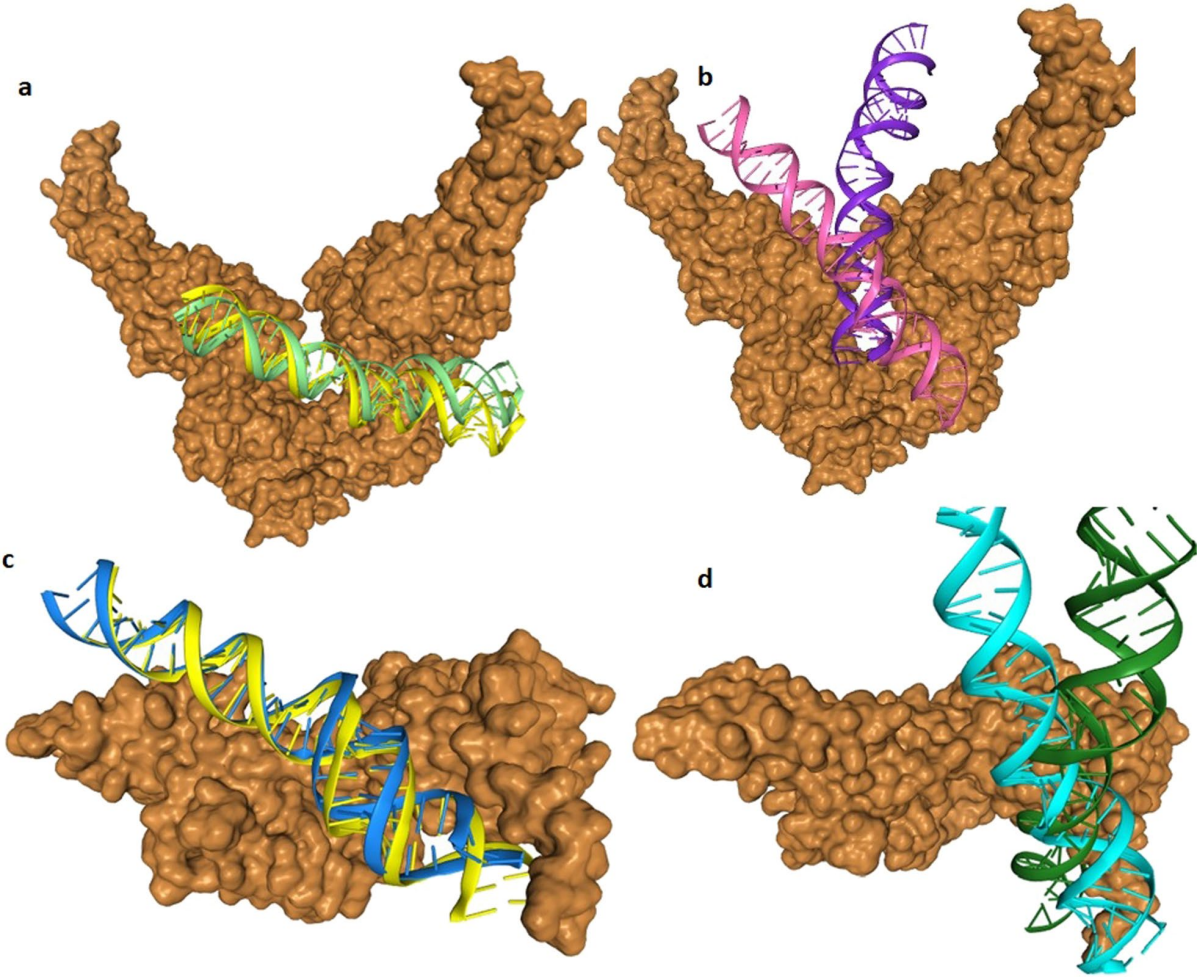
Molecular docking analysis of eNOS promoter with STAT3 and STAT6 transcription factor. **a** (WT) and **b** (mutant) represents the binding of STAT3 with *eNOS* promoter encompassing -786 T > C variant. Protein is shown in brown surface model while DNA in cartoon model. **c** (WT) and **d** (mutant) represents the binding of STAT6 with *eNOS* promoter encompassing -2015G > A variant. Protein is shown in brown surface model while DNA in cartoon model

### Docking analysis of eNOS promoter with STAT3 and STAT6

STAT3 and STAT6 protein's surface were assessed through molecular docking to investigate its potential for *eNOS* promoter region. Rigid body docking of *eNOS* DNA with STAT3 and STAT6 revealed important structural insight into the binding of DNA major and minor grooves. Mixture of major and minor grooves are involved in binding with respective transcription factor. Computational docking gave a perfect fit of binding of normal DNA with STAT3 and STAT6 protein while mutant promoter DNA moved away from the binding interface of both (STA3 and STAT6) transcription factor (Fig. 8).

## Discussion

PE is an important maternal health problem, especially in developing countries like Pakistan. Maternal Mortality is extremely high in Pakistan where 1 in 89 women dies of maternal causes with PE and eclampsia as one of the major causes [8]. Identification of various factors and candidate genes could help in understanding this complication and provide clues for its management and treatment. Genome wide association studies have been done on various population presenting the involvement of different genes and their variants [36–38]. In addition endothelial dysfunction, role of *eNOS* gene and reduced NO production has been associated with PE [11, 16, 39]. This study was the first to assess the potential role of NO levels and *eNOS* gene variants in preeclamptic women as compared to healthy normotensive pregnant women in Pakistan.

According to current study, maternal age body mass index (BMI), systolic and diastolic blood pressure, Gestational age between control and preeclamptic groups showed significant role in PE. Similar results were reported previously studies presenting that women with increased age and BMI had twice the risk of developing PE and increases up to 30% in every additional year of age past [40–42]. History of PE in previous pregnancy and family history of preeclampsia also predicts the association with the onset of PE. Family history of PE is associated with a fourfold increased risk of PE in women, underlying mechanism may involve the predisposition of various genetic factor involved in the pathophysiology of PE [41, 43]. Results of the current study revealed that levels of NO were significantly lower in preeclamptic group as compared to controls in accordance with previous findings [10, 44–46]. Due to its short half-life, the direct measurement of NO is extremely challenging in a complex biological environment. As NO is rapidly metabolized to nitrite and nitrate in the presence of oxygen, the determination of both nitrite and nitrate (termed NOx) is commonly used to estimate total NO production. A major general disadvantage of nitrite/nitrate determination to estimate tissue NO level is that dietary intake of nitrite (e.g. cured meat) and nitrate (e.g. vegetables) are rather significant, which markedly influences plasma NOx level [47]. The Griess reaction was used for the determination of NO in current study it is also called diazotization assay which is based on the conversion of nitrite to a purple-coloured azo-dye that can be spectrophotometrically assayed at a wavelength of ∼540 nm. Advantages of this method include a strong literature background, numerous commercially available reagent kits and wide availability of infrastructure [48]. It was evident previously that impair NO levels attenuated the acetylcholine-induced relaxation in arteries of preeclamptic women placentas causing vasoconstriction leading to increased mother’s blood pressure [49, 50]. Therefore, circulating NO is likely to be important for fetoplacental hemodynamics ensuring adequate placental blood flow and fetal oxygenation [17].

In present study common variants of *eNOS* gene including c.894G > T (p.(Glu298Asp)), intron 4b/4a and g.-786 T > C were analyzed by genotyping and confirmed through sequencing. The significant association was found for c.894G > T (p.(Glu298Asp)), variant. It was demonstrated that *eNOS* c.894 T (p.298Asp) was significantly associated with PE. Our findings are in correspondence with previous studies [1, 15, 18, 20]. A likely mechanism by which c.894 T (p.298Asp) might reduce the bioavailability of NO has been reported. Various studies have shown that p.298 Asp is subjected to selective proteolytic cleavage in vascular tissues and endothelial cells resulting in reduced vascular NO generation in subjects homozygous for this variant [20, 51, 52]. In addition, p.(Glu298Asp) modulates *eNOS* activity by interacting with other proteins involved in the degradation of its product, and hence its intracellular distribution [52]. Thus, women with homozygous Asp298 allele are more susceptible to endothelial dysfunction which might increase the risk of PE, observed in the present study as well.

Regarding the *eNOS* intron 4b/4a variant, non-significant association has been observed in present study as demonstrated by previously in different population-based case control studies [11, 16, 21, 53–55]. Possible reason might be its intronic region which is unlikely to be functional in its own right [15].

The association of g.-786 T > C variant with PE seen here agreed with the results established earlier for different regions of the world [21, 39, 53, 56, 57]. The present study demonstrated that g.-786C allele was prevailing in PE patients as compared to control. The g.-798C allele in the *eNOS* promoter region has been associated with reduced mRNA expression and lower serum nitrite/nitrate levels Causing unavailability of NO [58]. It was also suggested that 50% of *eNOS* activity can be reduced if its transcription is repressed by targeting g.-786C allele through single-stranded DNA binding transcription factor replication protein, A1 which results in lower NO production [59, 60]. Interestingly, the eNOS haplotype (‘C Glu a’) was also found in association with responsiveness to antihypertensive therapy in PE patients. It is possible that this particular eNOS haplotype leads to a lower NO availability in PE patients, which is improved by antihypertensive drugs. [61].

Sequencing revealed the association of another variant with the disease phenotype that is g.2051G > A which was present in the promoter region of the *eNOS* gene. Genetic analysis showed that previously this variant was associated with the disease phenotype in heterozygous form but in accordance with resent study it was also determined to be homozygous for g.2051AA. This variant might affect the transcription of the *eNOS* gene through unknown mechanism and results in reduced NO levels leading to endothelial dysfunction.

Through our in silico deep structural analysis, we elucidated the structural and functional behavior of *eNOS* protein upon Glu298Asp substitution. The reported substitution resulted in the loss of flexibility and deformities were reported that alter the conformation and configuration of the protein and ultimately decrease protein stability. Molecular dynamic simulation analysis revealed that due to Glu298Asp substitution there was alteration in protein structural behavior and fluctuations were observed in the loop region encompassing the mutation and this alteration might play a role in causing preeclampsia. Detailed illustration of a structural and functional behavior of *eNOS* uncovers underlying molecular mechanism and may aid in the development of a potent therapeutic drug. Secondly the mutations reported in the promoter region of the *eNOS* gene changed the binding of transcription factor STAT3 and STAT6 to the 5’ site of the *eNOS* gene. These reported variants might change the regulation of *eNOS gene* and responsible reduced NO level and leading to endothelial dysfunction.

## Conclusion

The present study revealed substantial association of c.894G > T (p.(Glu298Asp)) and g.-786 T > C variants while no association of intron 4b/4a variant in Pakistani population. Data from the current study suggest that there might be other risk variants of the *eNOS* gene (g.2051G > A and g.1861G > A) and lower levels of serum NO that confers in an increased risk of PE. The detailed computational investigation further confirmed the deformities and changes in protein flexibility upon Glu298Asp. These structural alterations might be associated with PE. Variants in the promoter region of the *eNOS* gene further validate the change in gene regulation for the onset of disease. Identification of key structural and functional features in eNOS protein and gene regulatory region might be used for designing specific drugs for therapeutic purpose.

### Future perspective

Intense care units for PE should in every city in Pakistan for prompt diagnosis of PE in community settings are necessary to ensure maternal and fetal well-being. Genetic testing and NO concentration in maternal serum could be used as a biomarker to predict PE for practicing clinicians, which could also help in interfering PE development in the early period in preeclamptic Pakistani women. Limitations of the current study include small sample size, no follow up and limited resources so, large study or meta-analysis and follow up studies are needed to define the contribution of *eNOS* variants and altered NO production in PE pathogenesis and associated features. Further collaborative research in PE may help in elucidating the contributory role of *eNOS* variants by performing genetic association studies and genome-wide association studies with adequate credibility. Understanding of etiology of PE and the role played by the differences in the environmental factors in developed and developing countries should be a research priority to prevent maternal and neonatal morbidity and mortality.

## Supplementary Information

**Additional file 1: Figure S1.** Pie chart showing percentage of subjects included in the study from different areas of Pakistan.

## Data Availability

All the data are contained in the manuscript.

## Abbreviations

PE: Preeclampsia
eNOS: Endothelial nitric oxide synthase
NO: Nitric oxide
SEM: Standard error of mean
