# Supplementary material for: Association of endothelial nitric oxide synthase gene variants with preeclampsia
Source: Reprod Health. 2021 Jul 28;18:163. doi: 10.1186/s12978-021-01213-9 (PMC8320179; doi:10.1186/s12978-021-01213-9)
Supplement: Supplementary file 1 — Additional file 1: Figure S1. Pie chart showing percentage of subjects included in the study from different areas of Pakistan. [file 12978_2021_1213_MOESM1_ESM.docx]

**Figure :** Pie chart showing percentage of subjects included in the study from different areas of Pakistan.
